# Editorial: Therapeutic and Diagnosis Target Discovery Based on Metabolomics

**DOI:** 10.3389/fphar.2022.893905

**Published:** 2022-04-04

**Authors:** Linsheng Liu

**Affiliations:** Department of Pharmacy, The First Affiliated Hospital of Soochow University, Suzhou, China

**Keywords:** metabolomics, diagnostic target discovery, therapeutic target discovery, biomarkers, precision medicine

With the completion of the human genome sequencing project, the functions of gene fragments have gradually been deciphered, ushering in the postgenome era, and many “omics” technologies have been developed. Transcriptomics, proteomics, and metabolomics have all become research hotspots in the medical and biological fields at this stage and have gradually been applied to every aspect of clinical research. Metabolomics was first formally proposed at the end of the last century, and is described as a quantitative analysis of all metabolites in an organism and a research method to determine the relative relationship between metabolites and physiological and pathological changes. It is an integral part of systems biology. Metabolomics facilitates the identification of biologically meaningful markers compared with other omics sciences, such as genomics, transcriptomics or proteomics, because far fewer metabolites are present in an organism than genes, mRNAs and proteins. Metabolomics has been the backbone of system-wide analyses of disease and medicine owing to the critical roles of metabolites in biological processes and the growing understanding of how the metabolome dynamically affects biological systems. Therefore, metabolomics has been gradually applied to all aspects of clinical research in the past decade, promoting the development of precision medicine, such as determining the prognosis of diseases, monitoring adverse drug reactions, and discovering diagnostic biomarkers.

Over the past 10 years, researchers have gradually conducted an increasing number of metabolomic studies on the pathogenesis of various diseases to identify the differentially abundant metabolites between diseased and normal individuals and thus identify biologically valuable diagnostic or therapeutic metabolites. These studies involve analyzing cells, animal models of diseases, and clinical patients with the NMR and MS analytical platforms. This research topic “*Therapeutic and Diagnostic Target Discovery Based on Metabolomics*” consists of 9 articles contributed by more than 71 authors in the fields of metabolic pathways of small-molecule endogenous substances in the treatment or diagnosis of some diseases. The topic revealed the mechanisms underlying the pharmacological interactions between metabolic targets and available intervention strategies that provide further insights into the treatment of these diseases.

Osteoporosis is a highly occult disease with no obvious symptoms or sensitive biomarkers, and many patients are only diagnosed after a fracture occurs. Deng et al. integrated untargeted metabolomics, lipidomics and targeted metabolomics to screen biomarkers for osteoporosis. Changes in metabolites in patients with osteoporosis suggested a disturbance in the bile acid metabolism pathway and the potential of using HCA as a biomarker for the early diagnosis of osteoporosis. As important active small-molecule compounds involved in the interaction between the body and the gut microbiota, bile acids are also a hot spot of current research. Hu et al. documented the effect of metformin on the gut microbiota and host metabolic profiles in STZ- and high-fat diet-induced type 2 diabetic rats using 16S rRNA sequencing and untargeted and targeted metabolomics assays. More genera in DM rats were regulated by metformin than by insulin. Several genera, metabolites and bile acids were found to be related to metformin and insulin treatments. Qu et al. clarified that aberrant activation of the secondary bile acid biosynthesis pathway increased the hydrophobicity of the bile acid pool that might subsequently promote metabolic disturbances and disease progression in mice subjected to chronic unpredictable mild stress using an untargeted metabolomics method. Due to many adverse effects of gestational diabetes mellitus, Raczkowska et al. conducted an exhaustive assessment of multiple biomarkers for the prediction and diagnosis of gestational glucose intolerance in two OGTT categories (high FPG levels with normal postglucose PG levels and normal FPG levels with high postglucose PG levels).

Lipids are also an important component of the metabolome. Zhi et al. used UHPLC-HRMS to acquire lipid profiles from patients with Wilson’s disease (WD) and their relatives (and a control group) to determine characteristic lipid profiles of patients with WD and identify potential diagnostic or therapeutic biomarkers for WD. The findings may provide valuable insights into identifying diagnostic and therapeutic biomarkers for Wilson disease. Changes in amino acids are the most common features detected in metabolomics studies. Sun et al. presented a GC/MS-based metabolomics method to profile the dynamic endogenous metabolic changes in the serum of mice at different time points after partial hepatectomy. Several amino acid and glucose metabolism pathways were dynamically altered during liver regeneration. They also used machine learning algorithms to identify potential metabolites that predict liver regeneration performance. Metabolomics is often also studied in conjunction with other omics techniques. Li et al. evaluated the effect of mesoporous silica nanoparticles (MSNs) on the liver using histopathology, metabolomics, proteomics and transcriptomics. MSNs administered i.v. substantially altered the levels of several metabolites involved in hepatic metabolism, oxidative stress and inflammation pathways, and the changes were more significant than those observed after oral administration.

Arjmand et al. conducted a review focusing on a clinically interesting issue regarding the treatment resistance of patients with cancer and proposed the molecular docking modeling method as a novel approach to target the metabolic pathways in cancer stem cells. Finally, as one of the most relevant approaches to investigate metabolic phenotypes, Emwas et al. presented a brief review of fluxomics research conducted in recent decades by discussing recent studies and common analytical tools.

In conclusion, the “*Therapeutic and Diagnostic Target Discovery Based on Metabolomics*” research topic highlights the importance of developing novel targets and biomarkers for the discovery of targets for the diagnosis and treatment of complex diseases.

